# Incidence of Cerebral Cavernous Malformation-Related Epilepsy in Children: A Single Center Survey

**DOI:** 10.7759/cureus.38178

**Published:** 2023-04-26

**Authors:** Masahiro Narita, Yosuke Miyairi, Mitsuo Motobayashi, Akihiro Chiba, Yuji Inaba

**Affiliations:** 1 Pediatric Neurology, Nagano Children’s Hospital, Azumino, JPN; 2 Neurosurgery, Nagano Children’s Hospital, Azumino, JPN; 3 Pediatric Neurology, Nagano Children's Hospital, Azumino, JPN

**Keywords:** incidence, children, cavernous malformation-related epilepsy, epilepsy, cavernous malformation

## Abstract

Introduction: Cerebral cavernous malformations (CCMs) are rare developmental cerebrovascular malformations. The risk of epilepsy is high in patients with CCMs, but the incidence of epilepsy has not been reported in a pure pediatric population. We herein present 14 pediatric cases of CCMs, including five with CCM-related epilepsy, and examine the incidence of CCM-related epilepsy in this pediatric population.

Methods: Pediatric patients with CCMs who visited our Hospital between November 1, 2001, to September 31, 2020, were retrospectively screened for inclusion, and 14 were enrolled.

Results: Fourteen enrolled patients were divided into two groups based on the presence or absence of CCM-related epilepsy. The “CCM-related epilepsy group” (n = 5) consisted of five males with a median age of 4.2 (range: 0.3-8.5) years at the first visit. The “non-epilepsy group” (n = 9) consisted of seven males and two females with a median age of 3.5 (range: 1.3-11.5) years at the first visit. The prevalence of CCM-related epilepsy at the time of the present analysis was 35.7%. Follow-up periods in CCM-related epilepsy and non-epilepsy groups were 19.3 and 24.9 patient-years, respectively: the incidence was 11.3% per patient-years. The frequency of seizures due to intra-CCM hemorrhage as the primary symptom was significantly higher in the CCM-related epilepsy group than in the non-CCM-related epilepsy group (p = 0.01). Other clinical characteristics, i.e., primary symptoms including vomiting/nausea and spastic paralysis, magnetic resonance imaging findings, including the number or maximum diameter of CCMs, cortical involvement, intra-CCM hemorrhage, and infratentorial lesions, surgical resection, and non-epileptic sequelae, such as motor disability and intellectual disability, did not significantly differ between the groups.

Discussion: The incidence of CCM-related epilepsy in the present study was 11.3% per patient year, higher than in adults. This discrepancy may be attributed to these studies including both adult and pediatric patients, whereas the present study examined a pure pediatric population. The presence of seizures due to intra-CCM hemorrhage as the initial symptom was a risk factor for CCM-related epilepsy in the present study. To elucidate the pathophysiology of CCM-related epilepsy or the reason for its higher incidence in children than in adults, further analyses of a large number of children with CCM-related epilepsy are warranted.

## Introduction

Cerebral cavernous malformations (CCMs) are rare developmental cerebrovascular malformations. Previous studies reported CCMs in 0.16-0.8% of an all-ages group and 0.2-0.6% of a pediatric population [1-5]. Childhood-onset CCMs account for 25-35% of all patients with CCMs [1,6,7]. The risk of epilepsy is higher in patients with CCMs than those with other cerebrovascular malformations. The incidence of epilepsy in a five-year follow-up was 94% in patients with CCMs and 58% in those with arteriovenous malformations [7]. Only one prospective study on 139 adult patients with CCMs set the endpoint as the incidence of CCM-related epilepsy; the five-year risk of first-ever seizures was 6% in 38 patients with CCMs with intracranial hemorrhage or a focal neurological deficit and 4% in 57 patients without any CCM-related symptoms [8]. Furthermore, previous studies reported that 40-90% of patients with CCMs developed epilepsy, and the risk of epilepsy was estimated to be 1.5-4.3% per patient-year; however, these studies did not target a pure pediatric population [7,9]. We herein present pediatric patients with CCMs, and examine the incidence of CCM-related epilepsy.

## Materials and methods

Patients

**Figure 1:**
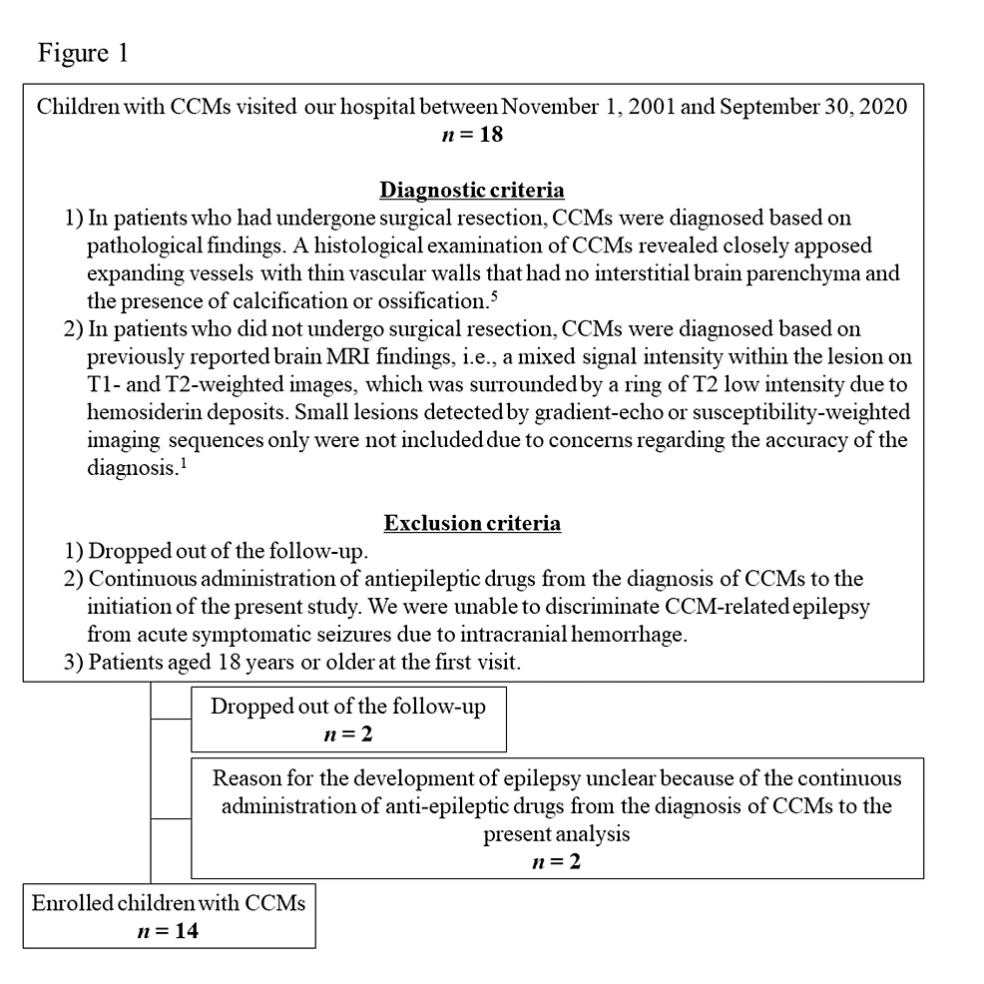
Flow chart of patient enrollment and exclusion. We screened 18 patients with cerebral cavernous malformations (CCMs) who visited our hospital. Four patients were excluded because they dropped out of the follow-up or were continuously administered antiepileptic drugs from the diagnosis of CCMs to the initiation of the present study. Therefore, 14 children were ultimately enrolled.

The present study was designed as a retrospective analysis at a single center. Pediatric patients with CCMs who visited Nagano Children’s Hospital between November 1, 2001, to September 31, 2020, were screened for inclusion, and 14 were ultimately enrolled. Diagnostic and inclusion criteria are shown in Figure 1. Namely, 1) in patients who had undergone surgical resection, CCMs were diagnosed based on pathological findings. A histological examination of CCMs revealed closely apposed expanding vessels with thin vascular walls with no interstitial brain parenchyma and calcification or ossification [10]. 2) In patients who did not undergo surgical resection, CCMs were diagnosed based on previously reported brain MRI findings, i.e., a mixed signal intensity within the lesion on T1- and T2-weighted images, which was surrounded by a ring of T2 low intensity due to hemosiderin deposits [1,11]. Small lesions detected by gradient-echo or susceptibility-weighted imaging sequences only were not included due to concerns regarding the accuracy of the diagnosis [1]. Exclusion criteria are as follows: 1) dropped out of the follow-up; 2) continuous administration of antiepileptic drugs from the diagnosis of CCMs to the initiation of the present study because we were unable to discriminate CCM-related epilepsy from acute symptomatic seizures due to intracranial hemorrhage; 3) patients aged 18 years or older at the first visit (Figure 1). The diagnosis of CCM-related epilepsy was established based on the following criteria: 1) epilepsy in patients with at least one CCM; 2) evidence of an epileptic focus close to CCM or a focal epileptic seizure that developed from the same hemisphere as CCM [10]. Patients who met the diagnostic criteria for known epileptic syndrome other than CCM-related epilepsy, such as self-limited focal epilepsy or idiopathic generalized epilepsy, were excluded [10].

Data collection

We used electronic medical records to retrospectively collect clinical information, including age, sex, primary symptom, brain MRI findings, surgical resection, and non-epileptic sequela. Two physicians confirmed the diameters of CCMs, and the average values were adopted.

Statistical analysis

Continuous data are presented as the median (range). The Mann-Whitney U test was used to assess the significance of differences in age at the onset or the last follow-up, the duration of follow-ups, and the number or maximum diameter of CCMs between the two groups. Statistical comparisons of categorized data were performed using Fisher’s exact probability test. All statistical analyses were conducted using Microsoft® Excel® for Windows version 2016, Statcel 4 software (OMS Publishing Inc., Saitama, Japan). The level of significance was defined as a p-value <0.05.

Ethics

The present study was approved by the Ethics Committee of Nagano Children’s Hospital (approval numbers: S-04-53 and 31-11).

## Results

Clinical characteristics

As shown in Table 1 and Figure 1, 14 patients were enrolled and divided into two groups based on the presence or absence of CCM-related epilepsy. The “CCM-related epilepsy group” (n = 5) consisted of five males with a median age of 4.2 (range: 0.3-8.5) years at the first visit. The “non-epilepsy group” (n = 9) consisted of seven males and two females with a median age of 3.5 (range: 1.3-11.5) years at the first visit. All these parameters were not statistically significant between the two groups.

Incidence of CCM-related epilepsy

The prevalence of CCM-related epilepsy at the time of the present analysis was 35.7%. Follow-up periods in CCM-related epilepsy and non-epilepsy groups were 19.3 and 24.9 patient-years, respectively: the incidence was 11.3% per patient-years.

A risk factor analysis of CCM-related epilepsy

The frequency of seizures due to intra-CCM hemorrhage as the primary symptom was significantly higher in the CCM-related epilepsy group than in the non-CCM-related epilepsy group (P = 0.01). Other clinical characteristics, i.e., primary symptoms including vomiting/nausea and motor disability, magnetic resonance imaging findings, including the number or maximum diameter of CCMs, cortical involvement, intra-CCM hemorrhage, and infratentorial lesions, surgical resection, and non-epileptic sequelae, such as motor disability and intellectual disability, did not significantly differ between the groups (Table 1).

**Table 1:** Clinical characteristics of patients with cavernous cerebral malformations (CCM) with or without CCM-related epilepsy and the incidence of CCM-related epilepsy. CCM: cavernous cerebral malformations

	CCM-related epilepsy group Median (range) or number	Non-epilepsy group Median (range) or number	P value	Incidence of CCM-related epilepsy
n	5	9		
Male/Female	5/0	7/2	0.40	
Age, years				
On the first visit	4.2 (0.3–8.5)	3.5 (1.3–11.5)	>0.05	
On the last visit	12.9 (5.0–18.0)	6.8 (4.3–14.8)	>0.05	
The total duration of the follow-up	9.5 (1.3–11.3)	2.3 (0.6–7.7)	>0.05	
Duration from the diagnosis of CCM to the onset of CCM-related epilepsy, years	0.8 (0.1–9.8)	–		
Follow-up period, patient-year	19.3	24.9		11.3% per patient-year
Primary symptom				
Seizure due to intra-CCM hemorrhage	4	2	0.01	
Vomiting/nausea	2	2	0.45	
Motor disability	2	2	0.45	
None	0	3	0.23	
Magnetic resonance image findings				
Number of CCMs	11 (1–27)	1 (1–28)	>0.05	
Solitary lesion	2	6	0.34	
≥10 lesions	3	1	0.09	
Maximum diameter of CCMs, mm	28.0 (16–38）	13.5 (5–48)	>0.05	
≥15 mm	5	7	0.13	
Cortical lesion	4	8	0.55	
Intra-CCM hemorrhage	5	9	0.71	
Infratentorial lesion	0	1	0.9	
Hemosiderin rim	5	9	0.71	
Surgical resection	3	5	0.34	
Non-epileptic sequela				
Motor disability	1	2	0.45	
Intellectual disability	1	1	0.60	
Seizure type				
Focal onset of seizures	5	–		

## Discussion

The incidence of CCM-related epilepsy in the present study was 11.3% per patient-year, higher than the previously reported estimate of 1.5-4.3% per patient-year [7,9]. This discrepancy may be attributed to these studies including both adult and pediatric patients, whereas the present study examined a pure pediatric population.

CCMs are generally not associated with epileptogenesis: neuronal damage to or inflammation of the surrounding brain parenchyma due to hemosiderin deposition, brain dysplasia, or CCM-associated hemorrhage may result in the development of epilepsy [12]. Previous studies, which included all age groups or only the adult population, identified the following established or controversial risk factors for CCM-related epilepsy: a supratentorial location, cortical involvement, mesial temporal localization, a high number of large CCMs, and a hemosiderin rim on MRI [10,13-17]. However, risk factors for CCM-related epilepsy in a pure pediatric population remain unknown. In the present study, seizures due to intra-CCM hemorrhage as the initial symptom were a risk factor for CCM-related epilepsy, consistent with previous findings on adults. We assumed seizures at the onset to reflect cortical involvement, followed by the development of epileptogenesis. Furthermore, the applicability of other established or controversial risk factors identified in mainly adult populations, i.e., a supratentorial location, cortical involvement, a high number of large CCMs, and a hemosiderin rim on MRI, was not confirmed in the present study. Unknown risk factors need to be identified in future large-scale studies on children.

The small sample size and retrospective study design limit the present results. Furthermore, the heterogeneity of reasons for diagnosing as CCMs could affect the results of the presented study. The non-epilepsy group contained 3 patients without symptoms associated with CCMs: they were diagnosed incidentally. This point could have an impact on the incidence of CCM-related epilepsy. To elucidate the pathophysiology of CCM-related epilepsy or the reason for its higher incidence in children than in adults, further analyses of a large number of children with CCM-related epilepsy are warranted.

## Conclusions

We herein present that the incidence of CCM-related epilepsy was 11.3% per patient-year in a pediatric population, and the presence of seizures due to intra-CCM hemorrhage as the initial symptom was a risk factor for CCM-related epilepsy. This is the first study to report the incidence of and risk factors for CCM-related epilepsy in a pure pediatric population.
